# Health Care Providers’ Trusted Sources for Information About COVID-19 Vaccines: Mixed Methods Study

**DOI:** 10.2196/33330

**Published:** 2021-12-08

**Authors:** Eden Brauer, Kristen Choi, John Chang, Yi Luo, Bruno Lewin, Corrine Munoz-Plaza, David Bronstein, Katia Bruxvoort

**Affiliations:** 1 School of Nursing University of California, Los Angeles Los Angeles, CA United States; 2 Department of Health Policy and Management Fielding School of Public Health University of California, Los Angeles Los Angeles, CA United States; 3 Department of Research & Evaluation Kaiser Permanente Southern California Pasadena, CA United States; 4 Southern California Permanente Medical Group Kaiser Permanente Southern California Pasadena, CA United States; 5 Department of Epidemiology School of Public Health University of Alabama at Birmingham Birmingham, AL United States

**Keywords:** health information, trust, health care provider, COVID-19, vaccine, mixed method, communication

## Abstract

**Background:**

Information and opinions shared by health care providers can affect patient vaccination decisions, but little is known about who health care providers themselves trust for information in the context of new COVID-19 vaccines.

**Objective:**

The purpose of this study is to investigate which sources of information about COVID-19 vaccines are trusted by health care providers and how they communicate this information to patients.

**Methods:**

This mixed methods study involved a one-time, web-based survey of health care providers and qualitative interviews with a subset of survey respondents. Health care providers (physicians, advanced practice providers, pharmacists, nurses) were recruited from an integrated health system in Southern California using voluntary response sampling, with follow-up interviews with providers who either accepted or declined a COVID-19 vaccine. The outcome was the type of information sources that respondents reported trusting for information about COVID-19 vaccines. Bivariate tests were used to compare trusted information sources by provider type; thematic analysis was used to explore perspectives about vaccine information and communicating with patients about vaccines.

**Results:**

The survey was completed by 2948 providers, of whom 91% (n=2683) responded that they had received ≥1 dose of a COVID-19 vaccine. The most frequently trusted source of COVID-19 vaccine information was government agencies (n=2513, 84.2%); the least frequently trusted source was social media (n=691, 9.5%). More physicians trusted government agencies (n=1226, 93%) than nurses (n=927, 78%) or pharmacists (n=203, 78%; *P*<.001), and more physicians trusted their employer (n=1115, 84%) than advanced practice providers (n=95, 67%) and nurses (n=759, 64%; *P*=.002). Qualitative themes (n=32 participants) about trusted sources of COVID-19 vaccine information were identified: processing new COVID-19 information in a health care work context likened to a “war zone” during the pandemic and communicating information to patients. Some providers were hesitant to recommend vaccines to pregnant people and groups they perceived to be at low risk for COVID-19.

**Conclusions:**

Physicians have stronger trust in government sources and their employers for information about COVID-19 vaccines compared with nurses, pharmacists, and advanced practice providers. Strategies such as role modeling, tailored messaging, or talking points with standard language may help providers to communicate accurate COVID-19 vaccine information to patients, and these strategies may also be used with providers with lower levels of trust in reputable information sources.

## Introduction

### Background

The rapid onset of the COVID-19 pandemic has created a secondary “infodemic” of health information challenges globally [1,2]. Health information about COVID-19 has proliferated in news media and social media (ie, web-based applications for creating or sharing content and social networking), and has rapidly evolved as scientists and public health professionals learned new information about the transmission and management of SARS-CoV-2 [3,4]. The real-time availability of new scientific and health information on COVID-19 has undoubtedly aided pandemic response but has also created information challenges for health care providers and the public in navigating misinformation, contradictions, and complexity [5]. Understanding how to effectively navigate a complex health information environment is an essential component of pandemic response for health care providers, who must apply changing information about the COVID-19 pandemic to practice.

### Prior Work

Despite growing reliance on the internet as a source of health information, many individuals still rely upon health care providers to learn new health information [6,7]. There is strong evidence that physicians, nurses, and other health care providers are among the most trusted entities for health information [8,9]. Although having up-to-date pandemic knowledge is essential for health care providers to educate the public, in the COVID-19 pandemic, health care providers are challenged to keep pace with ever-growing health information on SARS-CoV-2 and COVID-19 [5]. Although there is much literature on health care professionals as a trusted entity for health information among the public, including information about COVID-19 vaccines [8], less is known about who health care professionals themselves trust for health information.

How health care providers learn new COVID-19 information and convey that information to patients is especially important in regard to COVID-19 vaccines. Evidence suggests that health care provider opinions about vaccines and vaccine recommendations can affect patient decisions about vaccines [10-12]. Though nearly 70% of the US adult public has received at least one dose of a COVID-19 vaccine as of July 2021 [13] and more than 80% of health care providers have received a COVID-19 vaccine [14,15], vaccination levels vary substantially by locale, and there are still sizeable populations of adults that are unvaccinated. Health care providers have the potential to address barriers to COVID-19 vaccination and increase vaccine confidence as the US vaccination strategy shifts from mass vaccination to more traditional clinic-based administration of vaccines [16-18].

### Study Purpose

Given the high level of public trust in health care professionals for health information, the health information literacy of providers is essential for appropriate patient education and communication about COVID-19 vaccines. However, to date, there have been few studies about the specific sources that health care providers rely on to find trusted health information and how these sources affect their discussions about COVID-19 vaccines with patients. The purpose of this mixed methods study was to investigate which sources of information about COVID-19 vaccines are trusted by health care providers and how they communicate COVID-19 vaccine information with patients.

## Methods

### Design

This study used an explanatory-sequential mixed methods design with data from a web-based survey followed by qualitative interviews [19]. The study took place from March to May 2021 at Kaiser Permanente Southern California (KPSC), an integrated health system with approximately 15 hospitals, 235 clinics, and over 20,000 clinical employees. A one-time survey was sent to KPSC health care providers to assess COVID-19 experiences, COVID-19 perceptions including trusted sources of information, and demographics characteristics. We also conducted semistructured interviews using Rapid Assessment Procedures (RAP) for qualitative research [20,21] to further investigate health care provider perspectives on trusted sources of COVID-19 vaccine information. The study was approved by the KPSC Institutional Review Board, and all participants gave informed consent.

### Survey Procedures

KPSC health care providers were eligible to participate in the survey if they were actively practicing in the KPSC health system at the time of the survey and had access to a web-enabled device to complete the survey (phone, tablet, computer). We engaged leadership in medicine, nursing, and pharmacy to email the survey opportunity to their staff. Two reminder emails were sent from clinical leadership, and they were also provided with study flyers to post at hospitals and clinics.

### Survey Measures

#### Outcome Measures

The primary outcome was a survey item asking providers to select which sources of information they trusted for learning about COVID-19 vaccines among the following: government entities (local, state, or federal), their health system employer, mainstream news (television, print, radio), social media, personal contacts, physicians, and other, where participants could specify other sources with free text. The categories were not mutually exclusive, allowing respondents to select multiple sources.

#### Exposure Measures

The primary exposure was self-identified provider type (physician, advanced practice provider [Physician Assistant or Advanced Practice Registered Nurse], nurse [Registered Nurse or Licensed Vocational Nurse], pharmacist, and other). We also examined demographic and health history characteristics of the sample, including gender, age, race/ethnicity, and history of testing positive for COVID-19.

### Rapid Qualitative Assessment Procedures

Qualitative data were collected to further elucidate perspectives on COVID-19 vaccines among health care workers. As part of the survey, respondents had the option to indicate their willingness to be contacted for a follow-up interview. From those participants who volunteered to be contacted for an interview, we stratified potential interviewees by provider type (physician, pharmacist, nurse) and whether they had received a COVID-19 vaccine (yes/no). We then contacted 10 participants in each of these six groups (physician-acceptor, physician-decliner, pharmacist-acceptor, pharmacist-decliner, nurse-acceptor, nurse-decliner, 60 potential participants total) to ensure that interviews reflected a range of experiences and perspectives regarding COVID-19 vaccine confidence and hesitancy among providers. Interview participants were offered a small gift as an incentive for their time.

Interviews were conducted by authors KC and JC, who are experienced researchers with a background in conducting qualitative research and using semistructured interview guides. KC has a background in nursing and health services research, and as such, she conducted all interviews with nurses. JC has a background in public health and health services research and conducted all interviews with physicians and pharmacists.

Semistructured interviews with providers who either accepted or declined the COVID-19 vaccine were conducted using RAP [20,21]. An interview guide was developed with open-ended questions and probes about providers’ experiences with information about COVID-19; the vaccines; and how they receive, gather, and appraise various information sources. Perspectives on educational resources or other interventions that could be used to support vaccine confidence were also explored. Interviews were conducted by a member of the research team with experience in qualitative research, took place by telephone, and lasted approximately 15 to 30 minutes each. Interview data were digitally recorded and then transcribed for analysis and triangulation with survey data.

### Analysis

For quantitative survey data, we used chi-square tests to compare health care providers (physicians, physician assistants and nurse practitioners, pharmacists, nurses, others) by which sources of information they had indicated that they trusted. Analyses were conducted using R version 4.0.3 (R Foundation for Statistical Computing). We systematically analyzed the qualitative data using inductive thematic analysis [22-24]. A member of the research team reviewed the interview transcripts for data familiarization and generated codes with attached segments of data that were relevant to the research question. These codes were reviewed by study investigators, collapsed or broadened to ensure good fit with the data, and organized into themes and subthemes. To enhance credibility, the technique of member-checking was used, where stakeholder representatives from each provider group (nurses, physicians, pharmacists) reviewed and provided feedback about preliminary analyses. Themes were further refined to capture the most salient patterns in the data and then triangulated with quantitative data to gain deeper insight about providers’ experiences with COVID-19 and vaccine information.

## Results

### Sample Description

A total of 3164 potential participants opened the survey, 3052 verified eligibility and consented to the survey, and 2948 went on to complete the survey. The sample comprised 45.0% (n=1326) physicians, 40.2% (n=1184) nurses, 8.8% (n=259) pharmacists, and 5.7% (n=169) advanced practice providers (Table 1). The majority of respondents were female (n=2051, 69.6%) and White (n=1087, 36.9%) or Asian (n=1153, 39.1%). About 8% (n=240) of respondents reported a history of testing positive for COVID-19, and 1.9% (n=55) of the sample reported being currently pregnant. Among the total sample, 91.3% (n=2683) reported receiving at least one dose of a COVID-19 vaccine.

**Table 1:** Sample description.

Variable		Participants (N=2948), n (%)
**Age (years)^a^**		
	18-30	65 (2.2)
	31-40	811 (27.5)
	41-50	1027 (34.8)
	51-60	697 (23.7)
	61-70	313 (10.6)
	>70	34 (1.2)
**Gender**		
	Female	2051 (69.6)
	Male	891 (30.2)
	Other	6 (0.2)
**Provider type**		
	Physician	1326 (45.0)
	Advanced practice provider	169 (5.7)
	Pharmacist	259 (8.8)
	Nurse	1184 (40.2)
**Race/ethnicity**		
	White	1087 (36.9)
	African American/Black	98 (3.3)
	Hispanic/Latinx	340 (11.5)
	Asian	1153 (39.1)
	Native American/Alaskan/Hawaiian	18 (6.1)
	Multiple	167 (5.7)
	Other	85 (2.9)
**Ever had COVID-19**		
	Yes	240 (8.1)
	No	2536 (86)
	Unsure	172 (5.8)
Received at least one dose of a COVID-19 vaccine^b^		2683 (91.3)
**Plans to recommend COVID-19 vaccines to patients**		
	Will recommend	2203 (74.9)
	Will recommend if asked	593 (20.2)
	Unsure	99 (3.4)
	Will not recommend	47 (1.6)

^a^One participant did not provide information on age.

^b^Nine participants skipped this question.

### Survey Results: Comparison of Trusted Information Sources by Provider Type

The most trusted source of COVID-19 vaccine information across all health care provider types in our sample was government agencies (n=2513, 84.2% of the sample), followed by KPSC (n=2191, 74.3%). The least frequently trusted source of COVID-19 information by health care providers in our sample across all provider types was social media (n=691, 9.5%). When comparing information sources by provider type, there were significant differences for three information sources: government agencies, employer, and news media. More physicians trusted government agencies (n=1226, 93%) than nurses (n=927, 78%) or pharmacists (n=203, 78%; *P*<.001). For trust in one’s employer, there were differences for physicians compared with nurses and advanced practice providers. Although advanced practice providers trusted their employer at a frequency of 67% (n=95) and nurses at 64% (n=759), 84% (n=1115) of physicians reported trusting their employer for information about COVID-19 (*P*=.002). Overall trust in news as a source of information was lower for all provider groups (*P*=.003), but physicians (n=66, 27%) and pharmacists (n=351, 25%) more frequently reported trusting news media than advanced practice providers (n=29, 21%) or nurses (n=231, 20%). When compared to other provider groups, nurses generally reported lower levels of trust in nearly all information sources.

### Interview Results

A total of 32 interviews were conducted across all provider/vaccine groups (15 nurses, 8 pharmacists, 9 physicians). Of these, 17 interviewees indicated that they had declined the vaccine (10 nurses, 4 pharmacists, 3 physicians). In this analysis on experiences with information about COVID-19 and COVID-19 vaccines, we report on two overarching themes among provider vaccine acceptors and decliners: processing information in a health care work context likened to a “war zone” during the pandemic and communicating information to patients.

#### Theme: Processing Information in a Health Care Work Context Likened to a “War Zone”

The first theme reflects provider accounts of navigating the constant influx of new information during the COVID-19 pandemic while also managing fluctuating work demands and protecting their own health and safety in a workplace, described by several participants as a “war zone.” As one nurse-decliner stated:

It was absolutely horrible. Patients were dying every day. Lots and lots of death that I witnessed there, lots of strain on staff. Physically, mentally, it was hard.

The war zone work environment was characterized by unpredictability, with one nurse-decliner recalling, “it didn’t really seem like anyone knew what was going on,” while another nurse-decliner described work as “different every day.” Several providers recalled being unexpectedly “deployed” to COVID-19 units and having to adapt to rapidly changing information, patient volume and acuity, and work responsibilities. One nurse-decliner explained, “there was no warning, this was pandemic world.” Providers also observed the impact of these conditions on quality of care. One nurse-decliner stated, “there was no choice. [...] We couldn’t provide the same level of care.”

##### Subtheme: Valuing Transparency

Participants described how they evaluated COVID-19 and vaccine information in these circumstances. Many providers emphasized the need for transparency and “more balanced information,” particularly in the context of government and corporation-led dissemination, with one nurse-acceptor stating:

It would be helpful if [...] people knew that it wasn’t just these two [pharmaceutical] companies or the government that was supporting it.

Another nurse-decliner shared:

It’s very one-sided, the information that’s being given out. People have a false sense of security thinking they’re vaccinated because they don’t think they can still get COVID. It fits the narrative.

##### Subtheme: Acknowledging Ambiguity

Providers also questioned the oversimplification of COVID-19 information and vaccination decisions, with one nurse-decliner explaining, “It didn’t answer our doubts.” Part of this questioning stemmed from their firsthand experiences with unfolding information early in the pandemic. A nurse-decliner remembered:

Trying to preserve PPE [personal protective equipment], when we weren’t really sure how [the virus] was transmitted.

Many participants felt that oversimplification and lack of transparency contributed to feelings of hesitancy, distrust, or questioning. Instead, there was a preference for open acknowledgment of the complexities and limitations of available information, and respect for multiple points of view. As one pharmacist-acceptor pointed out, little attention was paid to “figuring out what those issues are [related to hesitancy] and addressing those issues.”

In making sense of COVID-19 information, participants also described the need to recognize the biases in their professional experiences. One nurse-decliner shared:

As healthcare workers sometimes our perspectives can be skewed, toward really bad. We’re not going to see people that have mild cases. I have to remind myself, “This isn’t what everyone is going through that has COVID.”

##### Subtheme: Appraising Various Sources of COVID-19 Information

**Figure 1:**
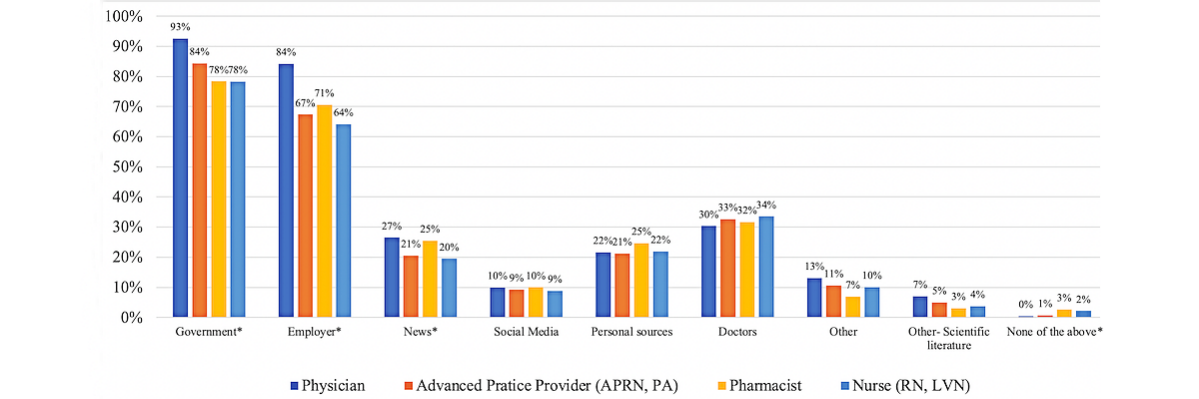
This figure shows the frequency of which sources of information health care providers (N=2948) reported trusting in a survey conducted from March to May 2021 in Southern California. *Group differences were significant at the .005 level in a chi-square test. APRN: advanced practice nurse; LVN: licensed vocational nurse; PA: physician assistant; RN: registered nurse.

Participants shared their perceptions of various sources of information related to COVID-19 vaccines. As displayed in Figure 1, nearly all providers identified major governmental entities such as the Centers for Disease Control and Prevention (CDC) as their primary source of trusted COVID-19 vaccine information, and this was generally consistent in interviews as well. However, a small subgroup of providers—most often, nurses—expressed misgivings about government sources during the interviews. As one nurse-decliner noted of information from the CDC:

It changes all the time so it’s really scary. It’s a lot of changes. It’s kind of hard to rely on data when data is practically new all the time.

Another nurse-decliner perceived changes in information and discrepancies with other organizations as reasons to distrust the CDC, stating:

I’m really not trusting what the CDC is saying, just because they have just been going back and forth...They’re contradicting what the World Health Organization is saying. I really question the FDA [Food and Drug Administration], I question the CDC.

One nurse-decliner appreciated the visual information provided at the county level by local government officials, explaining, “I’m very visual, I need to see the graphs, I need to see the trends.”

Participants also reported strong levels of trust in the information provided by their health system employer. Some described using updates from their employer as a reference in their clinical practice, but others noted the challenge of keeping up with the constant barrage of information from management. As one pharmacist-acceptor stated, “After a while, you keep posting things on the wall and it just ends up being wallpaper.”

Trust in mainstream news as an information source was low across all provider groups, with one nurse-decliner sharing, “I stopped watching the news.” Although overall trust in social media was comparable to mainstream news, some providers emphasized the credibility of personal testimonies, or what one nurse-decliner called “real life experiences, real life realities,” shared on such platforms. Another nurse-decliner used social media as an information starting point, explaining:

I definitely get most of my news from social media, from Instagram. Then I go research it for myself to make sure it’s true.

The public social media accounts of frontline physicians were also mentioned as trustworthy information sources.

In addition to the major sources of information listed, many participants described how they relied on their own personal experiences with COVID-19 as an information source in their perspectives about the vaccine. For some, the experience of personally becoming ill or caring for ill family members provided information about COVID-19 that was distinct from other conventional information sources, and these experiences influenced their perspectives on vaccine decision-making. One nurse-decliner said:

We actually ended up having COVID. I still can’t taste very well, I still can’t smell very well, I’m not 100% back to where my energy level was and that’s part of why I’m still hesitant to get the vaccine.

Another nurse-decliner shared:

I actually had COVID a few weeks ago, and my views on the vaccine have truly changed. It’s been rough. [...] I still feel congested, still have a mild cough, don’t have 100% energy.

In sharing about loved ones who had COVID-19, a physician-acceptor stated, “most everyone survived thankfully but I do have friends who still have symptoms.” Finally, some providers explained that no single source of information was sufficient in the context of rapidly evolving information, with one physician-acceptor stating, “for me, the key is to have multiple sources of information.”

#### Theme: Communicating Information About COVID-19 and Vaccines to Patients

The second overarching theme relates to how health care providers communicate COVID-19 and COVID-19 vaccine information to patients. Many participants described the impact of a changed work environment, specifically a shift to telehealth, which resulted in “limited face-to-face encounters with patients” and required new approaches to sharing information.

##### Subtheme: “Being a Role Model Matters”

Many participants who had received the COVID-19 vaccine believed it was their professional responsibility to serve as an example to patients. They described the impact of disclosing their own personal vaccine experiences in conveying information to patients. One nurse-acceptor explained:

When you see me, and I’m like, “Hey, I’m three months out from my second dose and I’m doing fine,” I’m a witness that it’s OK.

Similarly, a physician-acceptor stated:

I think it does help to say as a physician that I’ve been vaccinated and that it was fine for me and that I believe in it. Being a role model matters.

##### Subtheme: “Tailoring the Message”

Many participants recognized a need to “tailor the message” in communication with patients to reflect individual preferences and values. In some instances, this meant framing the risks of vaccines in the context of benefits; for example, focusing the discussion on serious risks such as hospitalization, death, and other long-term consequences. Tailoring the message also involved consideration of incentives that might resonate with an individual patient. In some cases, participants discussed the vaccine as a path back toward normalcy, with one physician-acceptor stating, “it can allow us to get back to normal life again, and that’s exciting.” For others, the health and safety of others—loved ones or the broader community—was used to invoke collective responsibility and an opportunity to help, particularly with patients who did not perceive COVID-19 as a serious threat to their own health. Tailoring the message was also important in preserving patients’ sense of autonomy in vaccine decision-making. One physician-acceptor explained, “these are the options, these are the pros and cons, take your pick.”

In tailoring vaccine messaging, providers discussed prioritizing some patients over others considering available vaccine safety data and perceived patient risk. One pharmacist-acceptor reflected on how they would discuss the vaccine with young women and stated:

I wouldn’t encourage them as much, especially to females who are of childbearing age, because I don’t want to recommend something that prevents them from having a child.

Another pharmacist-acceptor said, “for the young healthy crowd, I wouldn’t push it as much as the older group.”

##### Subtheme: Recognizing Social, Political, and Historical Factors

Recognizing broader contextual factors in COVID-19 vaccine communication was an important consideration for participants. Several providers emphasized the technical nature of COVID-19 information and challenges in communication with people who lacked foundational science knowledge. For example, a nurse-acceptor said:

I don’t think there’s enough information out there to explain to the medical staff, EVS [environmental services], housekeepers, people who aren’t knowledgeable in the science aspect. Even nurses, some nurses, they don’t understand what mRNA does.

Another nurse-acceptor reflected:

You have these highly educated physicians, but then you have people who aren’t as educated who don’t have as much resources to get the education. It should be fair and equal.

Concerns about political and historical reasons for not getting vaccinated were also raised by participants. One physician-acceptor shared:

Nurses that I’ve spoken to and tried to encourage vaccination, what I’m aware of is, there’s actually a history in the Philippines where Sanofi rolled out a kind of mandatory dengue vaccine, and I think the government profited off of it but many children died. And, so there’s a lot of pressure, people tell me, from their family or others that are still living in the Philippines not to be vaccinated.

There was recognition that groups with specific social, political, and historical meaning around vaccination would benefit from tailored communication approaches. Ultimately, many participants found it “very difficult to convince someone to do it if they truly do not believe in it,” in the words of a pharmacist-acceptor.

## Discussion

### Principal Results

In this mixed methods study of COVID-19 vaccine experiences and perceptions, we examined the information sources that health care providers use and trust, and how they have navigated the COVID-19 *infodemic* [1]. Providers generally trusted government health sources—specifically the CDC, noted in qualitative interviews—and the health system where they practiced. They had less trust in news media and social media. Though these patterns were consistent across provider types, we found small differences in trust by provider type. Nurses, pharmacists, and advanced practice providers had less trust in information from government sources, their employer, and the news compared with physicians. Qualitative interviews suggested that this mistrust stemmed from frequently changing and at times conflicting information about COVID-19 from the government, challenging and even traumatic pandemic working conditions, and perceiving COVID-19 vaccine information to be “one-sided” such that it did not fully resolve providers’ questions and doubts. These experiences and perceptions may reflect differences in pandemic working conditions by provider type, leading to differences between physicians and other providers. For example, some physician specialties were able to provide care via telehealth during the pandemic, while nurses had a direct patient-facing role and may have found changing or conflicting information difficult to integrate with a traumatic or stressful pandemic clinical context. Health care providers have been significantly challenged by keeping abreast of the latest understanding and guidance on COVID-19 clinical practice in the midst of misinformation, a high volume of new scientific information, and errors in or misunderstanding of the latest science [25,26]. Providers have faced the difficult task of integrating evolving, incomplete information into their practice while also needing to take immediate action for their patients and manage potential implications of information changes for their own personal health and safety [27].

Providers who had received a COVID-19 vaccine shared strategies for how they communicated information about the vaccines to patients but also recognized that convincing patients who did not believe in the vaccine was challenging. These strategies included role modeling the benefits and safety of the vaccine by disclosing their vaccination status as providers, tailoring the messaging to patient concerns, and recognizing structural forces that might contribute to vaccine hesitancy in specific demographic subgroups. Health care providers described the challenge of making sense of and sharing technical data with diverse groups of patients while avoiding oversimplification and confronting misinformation about vaccines in the public. Other strategies for vaccine messaging have been proposed based on principles of social, communication, and behavioral science, such as prosocial appeals, framing recommendations positively, and making strong or presumptive recommendations for vaccination [28]. Our findings suggest that health care providers are weathering the challenge of providing patients with accurate information about COVID-19 vaccines but also that additional support for clinicians may be needed from public health entities and health systems so that they are fully prepared with messaging and educational tools. This may include standard messaging strategies and patient educational tools that providers can tailor. Additionally, there may be a need for interventions to reinforce health care provider trust in reputable information sources to ensure that providers are prepared to give accurate, quality information to patients.

### Limitations

This study had strengths and limitations that should be considered in interpreting its findings. The study used mixed methods, which allowed us to explore health care provider perspectives on COVID-19 vaccine information in greater depth than a survey alone would allow. The sample was large and diverse, representing multiple provider types and race/ethnicities. Study limitations were the cross-sectional and self-report nature of the survey. The study used voluntary response sampling, which did not allow for determination of an exact response rate or number of potential participants reached, which may have oversampled providers with favorable views about COVID-19 vaccines. However, levels of vaccination reported by providers in our study are consistent with other similar surveys and national averages, suggesting that the sample was reasonably representative [14,15,29]. The sources of trusted information assessed in our survey were not necessarily exhaustive of all sources providers may rely upon, although we provided an option for providers to write in an *other* response for sources not included in the survey response list. Finally, qualitative results were intended to explore quantitative findings in greater depth within our sample and thus are not necessarily generalizable. With qualitative research, there is risk for interviewer bias in data collection and coding/analysis, but we attempted to mitigate these risks by using a consistent interview protocol and using a team approach coming to consensus about codes and themes.

### Conclusion

Scientific evidence on the prevention and treatment of COVID-19 has been changing rapidly since the onset of the pandemic in early 2020. Early in the pandemic, the World Health Organization (WHO) passed a resolution on the COVID-19 response that included the importance of managing the *infodemic* in controlling the COVID-19 pandemic [30]. The WHO called for the provision of reliable content and science-based data to the public, measures to counter misinformation, and prevention of information activities that undermined public health response. As the uncertainty of the pandemic and the politicization of vaccines continue, there is a need to, first, ensure that all health care providers receive accurate information from reputable information sources that they can trust and, second, to ensure that health care providers have informational tools available to give quality information and recommendations to patients about vaccines.

## Abbreviations

CDC: Centers for Disease Control and Prevention
CIRT: Care Improvement Research Team
EVS: environmental services
FDA: Food and Drug Administration
KPSC: Kaiser Permanente Southern California
PPE: personal protective equipment
RAP: Rapid Assessment Procedures
WHO: World Health Organization
